# 

**Published:** 1895-10

**Authors:** 


### BOOKS RECEIVED.

Physical and Natural Therapeutics. The Remedial Use of Heat, Electricity, Modifications of Atmospheric Pressure, Climates and Mineral Waters. By Georges Hayem, M. D., Professor of Clinical Medicine in the Faculty of Medicine of Paris. Edited with the assent of the author, by Hobart Amory Hare, M. D., Professor of Therapeutics in the Jefferson Medical College of Philadelphia. In one handsome octavo volume of 414 pages, with 113 engravings. Cloth, $3.00. Philadelphia; Lea Brothers & Co., Publishers. 1895.

Pathology and Morbid Anatomy. By T. Henry Green, M. D., Lecturer on Pathology and Morbid Anatomy at Charing-Cross Hospital Medical School, London. Seventh American from the eighth and revised English edition. Octavo volume of 595 pages, with 224 engravings and a colored plate. Cloth, $2.75. Philadelphia: Lea Brothers & Co., Publishers. 1895.

Disorders of the Sexual Organs in the Male. By Eugene Fuller, M. D., Instructor in Venereal and Genito-Urinary Diseases, New York Post-Graduate Medical School. In one very handsome octavo volume of 238 pages, with twenty-five engravings and eight full-page plates. Cloth, $2.00. Philadelphia: Lea Brothers & Co., Publishers. 1895.

The Urine in Health and Disease and Urinary Analysis, Physiologically and Pathologically Considered. By D. Campbell Black, M. D., L. R. C. S., Professor of Physiology, Anderson College Medical School. In one 12mo volume of 256 pages, with seventy-three engravings. Cloth, $2.75. Philadelphia: Lea Brothers & Co., Publishers. 1895.

A Text-Book of Practical Therapeutics, with Especial Reference to the Application of Remedial Measures to Disease and their Employment upon a Rational Basis. By Hobart Amory Hare, M. D., Professor of Therapeutics and Materia Medica in the Jefferson Medical College of Philadelphia. With special chapters by Drs. G. E. de Schweinitz, Edward Martin and Barton C. Hirst. New (fifth) edition, thoroughly revised. In one octavo volume of 740 pages. Cloth, $3.75; leather, $4.75. Philadelphia: Lea Brothers & Co., Publishers. 1895.

A System of Surgery. In a series of Contributions by twenty-five English Authors. Edited by Frederick Treves, F. R. C. S., Surgeon to, and Lecturer on, Surgery at the London Hospital, Examiner in Surgery at the University of Cambridge. In two large and handsome octavo volumes. Volume I., 1178 pages, 463 engravings and two colored plates. Cloth, $8.00. Volume II., preparing. Philadelphia: Lea Brothers & Co., Publishers. 1895.

A Manual of Organic Materia Medica; Being a Guide to Materia Medica of the Vegetable and Animal Kingdoms. For the use of Students, Druggists, Pharmacists and Physicians. By John M. Maisch, Ph. D., Professor of Materia Medica and Botany in the Philadelphia College of Pharmacy. New (sixth) edition, thoroughly revised, by H. C. C. Maisch, Ph. G. In one very handsome 12mo volume of 509 pages, with 285 engravings. Cloth, $3.00. Philadelphia: Lea Brothers & Co., Publishers. 1895.

A Manual of Obstetrics. By A. F. A. King, M. D., Professor of Obstetrics and Diseases of Women in the Medical Department of the Columbian University, Washington, D. C., and in the University of Vermont, etc. New (sixth) edition. In one 12mo volume of 532 pages, with 221 illustrations. Cloth, $2.50. Philadelphia: Lea Brothers & Co., Publishers. 1895.

Index-Catalogue of the Library of the Surgeon-General's Office, United States Army. Authors and Subjects. Together with an Alphabetical List of Abbreviations of Titles employed in the Index-Catalogue. Volume XVI. W—Z Y T H U S. Washington: Government Printing Office. 1895.

Transactions of the Medical Society of the State of New York for the Year 1895. Published by the Society. 1895.

Lectures on Appendicitis and Notes on other Subjects. By Robert T. Morris, A. M., M. D., Fellow of the New York Academy of Medicine, American Association of Obstetricians and Gynecologists, etc., etc.

Octavo, pp. xviii.—163. With illustrations by Henry Macdonald, M. D. New York: G. P. Putnam's Sons, 27 West Twenty-third street. 1895.

Clinical Lectures on Diseases of the Nervous System. Delivered at the National Hospital for the Paralysed and Epileptic, London. By W. R. Gowers, M. D., F. R. S., Physician to the Hospital; Consulting Physician to University College Hospital, etc. Octavo, pp. 279. Price, $2.00. Philadelphia: P. Blakiston, Son & Co., 1012 Walnut street. 1895.

Some Physiological Factors of the Neuroses of Childhood. By B. K. Rachford, M. D., Professor of Physiology and Clinician to Children's Clinic, Medical College of Ohio, etc., etc. Duodecimo, pp. viii. —122. Price, $1.00. Cincinnati: The Robert Clarke Company. 1895.

Eyesight and School Life. By Simeon Snell, F. R. C. S., Ed., Ophthalmic Surgeon to the Sheffield General Infirmary and to the School for the Blind; Lecturer on Diseases of the Eye at the Sheffield Medical School; Consulting Ophthalmic Surgeon to the Rotherham Hospital. Duodecimo, pp. 77. With numerous illustrations. Price, 63 cents. Bristol: John Wright & Co. 1895.

A Text-Book on Nervous Diseases. By American Authors. Edited by F. X. Dercum, M. D., Clinical Professor of Diseases of the Nervous System in the Jefferson Medical College, Philadelphia. In one handsome octavo volume of 1052 pages, with 341 engravings and seven colored plates. Cloth, $6.00; leather, $7.00. Philadelphia: Lea Brothers & Co., Publishers. 1895.

